# Risk factors for poor health-related quality of life in patients with colon cancer include stoma and smoking habits

**DOI:** 10.1186/s12955-021-01850-5

**Published:** 2021-09-10

**Authors:** Catarina Tiselius, Andreas Rosenblad, Eva Strand, Kenneth Smedh

**Affiliations:** 1 Department of Surgery, Västmanland Hospital Västerås, Västerås, Sweden; 2 Centre for Clinical Research Västerås, Uppsala University, Västmanland Hospital Västerås, Västerås, Sweden; 3 Department of Medical Sciences, Uppsala University, Uppsala, Sweden; 4 Regional Cancer Centre Stockholm-Gotland, Stockholm, Sweden

**Keywords:** Colon cancer, Health-related quality of life, Stoma, Smoking, Risk factors

## Abstract

**Background:**

Previous studies have shown that health-related quality of life (HRQoL) is associated with the prognosis of cancer patients. The aim of this study was to investigate risk factors for poor HRQoL in patients with colon cancer.

**Methods:**

This was a prospective population-based study of patients with colon cancer included between 2012 and 2016. HRQoL was measured using the cancer-specific European Organization for Research and Treatment of Cancer Quality of Life Questionnaire C30. Multiple linear regression analysis adjusted for age, sex, body mass index, smoking habits, American Society of Anesthesiologists physical status classification, emergency/elective surgery, resection with/without a stoma and tumour stage was used.

**Results:**

A total of 67% (376/561) of all incident patients with colon cancer (196 [52.1%] females) was included. Mean (range) age was 73 (30–96) years. Patients with worse health (American Society of Anesthesiologists physical status 3 and 4), those with higher body mass index, smokers and those planned to undergo surgical treatment with a stoma were at a higher risk for poor HRQoL than the other included patients at baseline and 6-month follow-up.

**Conclusions:**

Patient characteristics such as smoking, high body mass index and worse physical health as well as treatment with a stoma were associated with lower HRQoL. Health care for such patients should focus on social and lifestyle behavioural support and stoma closure, when possible.

*Trial registration*: ClinicalTrials.gov (NCT 03910894).

## Background

Over the years surgical and oncological treatments have improved in patients with colorectal cancer [1, 2]. However, a diagnosis of colorectal cancer has a major impact on the lives of patients. They experience functional impairments and other adverse effects related to the cancer. Therefore, it is important to assess these patients and identify the major predictors of HRQoL.

The term ‘HRQoL’ is multifactorial and subjective [3], and therefore difficult to quantify. The definitions of health and quality of life by the World Health Organization include information regarding the patient’s perceived physical, emotional and social functioning, which together can be labelled as HRQoL. The well-validated European Organization for Research and Treatment of Cancer Quality of Life C30 (EORTC QLQ-C30) generic questionnaire has been developed to capture cancer-specific symptoms and is widely used [4].

Several factors are known to be associated with HRQoL in patients with colorectal cancer, such as socio-demographic characteristics, treatment-related factors and lifestyle-related factors such as smoking, physical activity, diet and alcohol intake [5, 6].

The impact of early and advanced colon cancer in terms of HRQoL is probably different. Therefore, disease stage should be considered, and long-term studies on HRQoL are important. In patients with advanced disease, fatigue appears to be the most damaging factor [7]. In addition, patient-related factors such as age are important. Long-term follow-up studies from Germany and the Netherlands that used the EORTC QLQ-C30 questionnaire reported that younger patients have more adverse symptoms than older patients [8, 9]. A study on cancer survivors has shown that these patients experience unmet needs regarding a variety of different psychosocial factors [10]. Moreover, the perception of disease and coping abilities are important [11]. Different treatment strategies might also have an impact on HRQoL. By identifying these risk factors, targeted early intervention and the development of methods to facilitate rehabilitation could be possible, which in turn could enhance patients’ HRQoL.

Studies on HRQoL have been performed in patients with colorectal cancer in several countries at the national level, but no data are available for patients with colon cancer in Sweden. Therefore, this study aimed to investigate the HRQoL in a well-defined population of patients with colon cancer at diagnosis and at 6 months of follow-up using the EORTC QLQ-C30 questionnaire. In addition, we also aimed to compare these data with those of a general Swedish reference population [12] to determine whether there were differences in HRQoL between these populations. Furthermore, we sought to identify risk factors associated with poor reported outcomes for HRQoL in patients with colon cancer at diagnosis and 6-month follow-up.

## Methods

### Study population

**Fig. 1:**
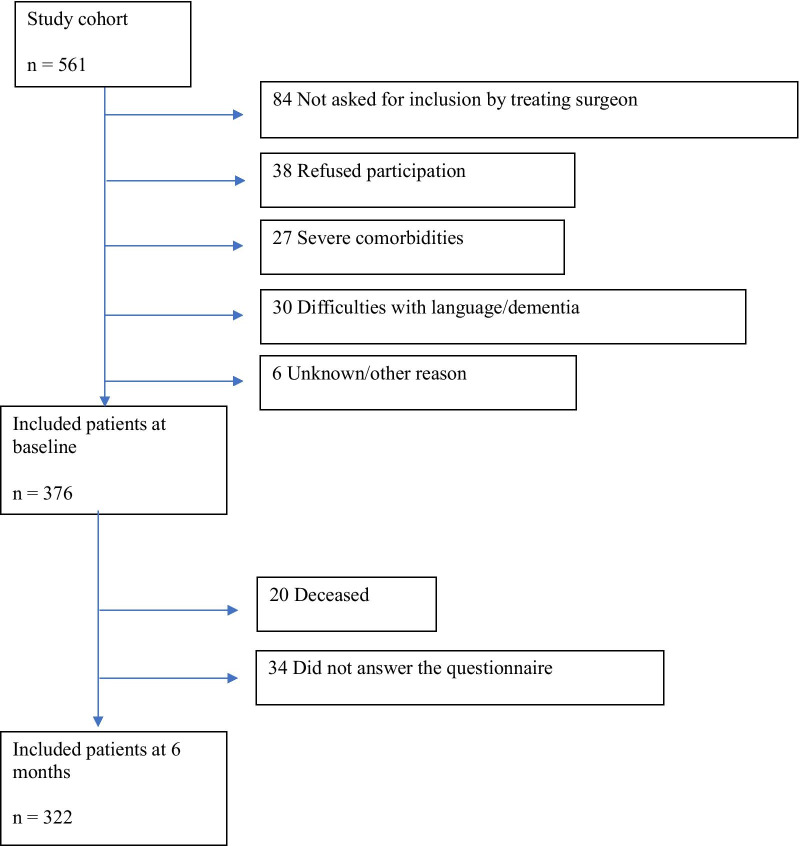
Flow chart of study patients diagnosed with colon cancer at baseline and at the 6-month follow-up

This was a prospective cohort study comprising all patients diagnosed with colon cancer between March 2012 and September 2016 at Västmanland Hospital Västerås, Sweden. Västmanland is a medium-sized Swedish county located approximately 100 km west of Stockholm with approximately 270,000 inhabitants. Västmanland Hospital Västerås is the only hospital in the county that provides treatment to patients with colon cancer. Västmanland is considered representative of Swedish society because of its distribution of educational, income and employment levels, as well as urban and rural areas [13]. In total, 376 patients were enrolled in the study, representing 67% (376/561) of all incident colon cancer cases in the county during that time period (Fig. 1). All included patients provided written informed consent to participate in the study and were guaranteed confidentiality. They were included in the ward or outpatient clinic by the colorectal surgeon. At the 6-month follow-up, 20 patients had died while 34 did not return the questionnaire. The exclusion criteria were an inability to understand the questionnaire or severe comorbidity.

This study was approved by the Regional Ethics Committee of Uppsala University (Dnr 2011/417) and registered at ClinicalTrials.gov (NCT 03910894).

### Measurements

#### Demographic and tumour stage characteristics

Clinical data were collected from the patient, radiologist, anaesthesiologist and pathologist, and were recorded in a local research protocol by the colorectal surgeon. Body mass index (BMI) was calculated as an individual’s weight in kg divided by their height in meters squared (kg/m^2^). The American Society of Anesthesiologists (ASA) physical status classification (1–4) was used as a proxy of comorbidity. We compared patients with better health (ASA status 1 and 2) with patients with worse health (ASA status 3 and 4). Pathological staging (tumour–node–metastasis, TNM; I–IV) was recorded. We compared patients with metastases (TNM IV) with patients with non-metastatic disease (TNM I–III).

#### Smoking status

Patients were asked about their smoking habits (non-smoker, former smoker or current smoker). A former smoker was defined as having smoked during the previous 10 years. We compared current smokers with former and non-smokers.

#### Treatment

Both surgically and non-surgically treated patients were included. Patients entered the hospital through the emergency ward or were referred to the outpatient clinic (elective). Patients who were treated surgically underwent a right, left or total colectomy with (stoma with resection) or without a stoma. Left-sided surgery included left, sigmoid and high-anterior resections. One group of patients was treated only with a stoma (stoma without resection) because of severe comorbidity or metastatic disease. Patients with TNM stage III disease who underwent postoperative chemotherapy received treatment for 3 months as routine therapy. Moreover, patients with TNM stage IV disease could have ongoing palliative treatment.

### Data collection

The data on demography and clinical variables were collected prospectively before treatment by the surgeon using a predefined local quality registry form, and then compared with data from the Swedish Colorectal Cancer Registry, which served as the control data. Decisions about treatment for individual patients were made in multidisciplinary meetings and followed the processes of the National Colorectal Cancer follow-up program. All patients in this study agreed to be included in the local and national registries.

Patients who agreed to participate in the HRQoL study provided written informed consent and were asked to complete the EORTC QLQ-C30 questionnaire at diagnosis and at the 6-month follow-up (Fig. 1). The time window to complete the baseline form was within a month after diagnosis and before surgery or at the start of palliative treatment. At the 6-month follow-up, the HRQoL questionnaire was sent to all participating patients. Non-responders were mailed up to two reminder letters.

### HRQoL questionnaire

The EORTC QLQ-C30 is a HRQoL generic questionnaire developed by the EORTC Quality of Life Study Group to assess QoL in patients with cancer in clinical trials [14]. It has been validated in both cancer and in general population. It consists of 30 items comprising five functional scales (physical, role, emotional, cognitive and social) and three symptom scales (fatigue, nausea and vomiting, and pain). Six single items are also included (dyspnoea, insomnia, appetite loss, constipation, diarrhoea and financial difficulties). The final two items in the questionnaire assess global health and overall QoL. Raw data were transformed to standardized scores ranging from 0 to 100 using the instructions of the EORTC scoring manual version 3.0 [15]. A high score on the functional scales represents a high level of functioning (i.e. a higher score is better), whereas a high score on the symptom scales represents a high level of symptoms (i.e. a lower score is better). Differences in mean QoL scores > 10 points were considered clinically meaningful [16].

#### General reference population

The population reference values used in this study were retrieved from a Swedish reference study including a total of 3069 age-stratified individuals, born between 1918 and 1979, who completed the EORTC QLQ-C30 questionnaire version 3.0. These individuals were selected from a population-based registry (SEMA) and represented a random sample of the general population in Sweden [12].

### Statistical methods

Categorical data are presented as frequencies and percentages, n (%), while ordinal and continuous data are presented as means with accompanying standard deviations. Pearson’s χ^2^ test and Welch’s independent samples *t*-test (because the population variances were not assumed to be equal) were used for categorical and continuous variables, respectively, to evaluate the significance of differences in demographics, lifestyle and clinical risk factors between included and non-included patients. Tests of differences in HRQoL between the participating individuals and the Swedish reference population were performed using Welch’s independent samples *t*-test separately for men and women. Aggregated sex-specific EORTC QLQ-C30 data for individuals aged 70–79 years reported by Michelson et al. [12] were used as reference (standard) values separately for men and women. Tests of differences between participants’ data at baseline and follow-up were performed using a paired samples *t*-test.

The associations between demographics, lifestyle and clinical risk factors (independent variables) and the change in HRQoL from baseline to follow-up (dependent variable) were examined using separate complete-cases multiple linear regression analyses for each EORTC QLQ-C30 scale/item. Male sex, age (years), smoking, BMI (kg/m^2^), ASA classification 1 or 2 (patients with better health), emergency surgery, TNM stages I–III (patients with non-metastatic disease) [reference: TNM stage IV] and stoma (with resection, without resection or no stoma [reference]) were included as independent variables in the multiple regression analyses, together with the baseline values of the EORTC QLQ-C30 scale/item in question. Results from the linear regression analyses are presented as the β coefficient (i.e. the coefficient of the slope indicating the magnitude of the linear association between the independent and dependent variables) with accompanying 95% confidence intervals (CIs).

All statistical analyses were performed using IBM SPSS Statistics software (v. 24 or later; IBM, Armonk, NY, USA), with *p* values < 0.05 considered statistically significant.

## Results

The baseline characteristics of the colon cancer study population are presented in Table 1. Data of patients who were included in the HRQoL study (n = 376) and those who were not included (n = 185) are presented. No major differences were found between included and non-included patients with respect to age, sex, smoking status or BMI. The non-included patients had worse health and a more advanced tumour stage (TNM). More included patients had undergone elective surgery and had better health (lower ASA status), and fewer such patients received a stoma, compared with the non-included patients. In total, 67/376 (18%) patients entered the hospital through the emergency ward. Of these, 59 underwent emergency surgery. Of the patients receiving emergency surgery, 31/59 (52%) and 10/59 (17%) were operated with a stoma, with and without bowel resection, respectively.

**Table 1:** Descriptive characteristics for patients included in the HRQoL study group (n = 376) and those who were not included but were registered in the local and national protocol (n = 185)

Variable	Included (n = 376)		Not included (n = 185)		*P* value
	Value	Missing, n (%)	Value	Missing, n (%)	
Male sex, n (%)	180 (47.9)	0 (0.0)	89 (48.1)	0 (0.0)	0.858^a^
Age (years), mean (SD)	73.3 (10.9)	0 (0.0)	73.6 (13.2)	0 (0.0)	0.777^b^
Current smoker, n (%)	34 (9)	16 (4.3)	12 (6.5)	55 (29.7)	0.943^a^
BMI (kg/m^2^), mean (SD)	26.6 (4.4)	4 (1.1)	25.8 (5.6)	11 (5.9)	0.120^b^
ASA status, n (%)		0 (0.0)		4 (2.2)	**< 0.001**^a^
1	43 (11.4)		10 (5.5)		
2	185 (49.2)		76 (42.0)		
3	134 (35.6)		78 (43.1)		
4	14 (3.7)		17 (9.4)		
Operated, n (%)	343 (91.2)	0 (0.0)	135 (73.0)	0 (0.0)	**< 0.001**^a^
Emergency surgery, n (%)	59/343 (17.2)	0 (0.0)	80/135 (59.2)	0 (0.0)	**< 0.001**^a^
Operation type		0 (0.0)		0 (0.0)	**< 0.001**^a^
No resection	16/343 (4.7)		25/135 (18.5)		
Right-sided operation	191/343 (55.7)		59/135 (43.7)		
Left-sided operation	117/343 (34.1)		36/135 (26.7)		
Colectomy	19/343 (5.5)		14/135 (10.4)		
Laparotomy	0/343 (0.0)		1/135 (0.7)		
Stoma		0 (0.0)		0 (0.0)	**< 0.001**^a^
Yes, with resection	89/343 (25.9)		51/135 (37.8)		
Yes, without resection	14/343 (4.1)		28/135 (20.7)		
No	240/343 (70.0)		56/135 (41.4)		
TNM stadium, n (%)		0 (0.0)		1 (0.5)	**< 0.001**^a^
1	37 (9.8)		20 (10.8)		
2	155 (41.2)		45 (24.3)		
3	131 (34.8)		39 (21.6)		
4	53 (14.1)		79 (42.7)		
x			1 (0.6)		

Sums of percentages may differ from 100 due to rounding

*P* values from tests of differences between included and not included individuals using ^a^Pearson’s χ^2^ test and ^b^Welch’s independent samples *t*-test. Significant *P* values are given in bold

*ASA* American Society of Anesthesiologists, *BMI* body mass index, *HRQoL* health-related quality of life, *SD* standard deviation, *TNM* tumour–node–metastasis

Table 2 presents a comparison of HRQoL of the included patients with colon cancer with that of a Swedish reference population [10]. In the patients with colon cancer at baseline, lower functional scores were observed in 3/5 and 4/5 scales and higher symptom scores in 6/9 and 5/9 items of HRQoL for men and women, respectively, compared with the Swedish reference population. At the 6-month follow-up, men had improved constipation scores and women had improved role and emotional function and improved constipation scores compared with their respective sex in the Swedish reference population (Table 2).

**Table 2:** Comparison of the included colon cancer patient´s HRQoL with the Swedish reference population (Standard) at baseline and 6-month follow-up, univariate analyses. Data for men and women presented separately

Men		Standard^a^		Baseline			Follow-up			
Scale	n	Mean (SD)	n	Mean (SD)	P_standard_^b^	n	Mean (SD)	P_standard_^c^	n^d^	P_diff_^e^
Global health status	230	76.4 (22.8)	177	59.7 (23.6)	**< 0.001**	132	68.0 (22.4)	**0.001**	130	**0.008**
*Functional scales*										
Physical functioning	224	81.6 (22.7)	178	79.0 (20.8)	0.232	130	80.5 (18.5)	0.629	129	0.083
Role functioning	224	82.6 (29.7)	178	67.2 (34.7)	**< 0.001**	130	72.7 (31.4)	**0.004**	129	0.577
Emotional functioning	223	88.2 (17.3)	178	74.7 (21.1)	**< 0.001**	132	81.1 (22.3)	**0.002**	131	**0.006**
Cognitive functioning	228	85.2 (16.9)	178	85.3 (17.3)	0.954	132	83.1 (20.4)	0.313	131	0.074
Social function	230	89.1 (21.1)	178	77.9 (25.0)	**< 0.001**	132	73.3 (28.9)	**< 0.001**	131	**0.020**
*Symptom scales/items*										
Fatigue	227	21.5 (23.1)	178	39.0 (25.7)	**< 0.001**	132	33.7 (24.0)	**< 0.001**	131	0.350
Nausea and vomiting	234	2.5 (10.0)	178	9.5 (18.4)	**< 0.001**	132	5.6 (12.5)	**0.017**	131	0.527
Pain	226	19.2 (25.0)	178	23.0 (26.9)	0.144	132	14.8 (22.2)	0.084	131	**0.013**
Dyspnoea	232	23.7 (29.4)	178	29.2 (29.8)	**0.062**	130	27.4 (26.4)	0.216	129	0.525
Insomnia	231	11.8 (22.5)	178	26.7 (29.7)	**< 0.001**	132	21.0 (29.8)	**0.002**	131	**0.017**
Appetite loss	233	2.7 (11.9)	178	21.2 (32.2)	**< 0.001**	132	10.9 (23.8)	**< 0.001**	131	**0.036**
Constipation	234	6.7 (17.7)	178	12.8 (25.6)	**0.007**	131	6.4 (16.6)	0.855	129	**0.049**
Diarrhoea	231	4.2 (13.1)	177	24.1 (31.2)	**< 0.001**	131	18.6 (24.9)	**< 0.001**	130	0.422
Financial difficulties	230	5.4 (16.0)	175	6.5 (18.5)	0.539	132	8.8 (23.6)	0.138	129	**0.015**

Women		Standard^a^		Baseline Vås			Follow-up			
Scale	n	Mean (SD)	n	Mean (SD)	P_stand_^b^	n	Mean (SD)	P_stand_^c^	n^d^	P_diff_^e^
Global health status	285	69.9 (25.0)	192	59.2 (22.9)	**< 0.001**	157	66.5 (23.7)	0.152	153	**0.001**
*Functional scales*										
Physical functioning	283	74.2 (23.6)	190	72.0 (21.0)	0.296	157	73.6 (20.9)	0.779	151	0.477
Role functioning	280	80.4 (29.6)	191	67.9 (32.6)	**< 0.001**	156	72.8 (29.7)	**0.010**	151	0.497
Emotional functioning	277	80.4 (22.0)	192	69.0 (22.6)	**< 0.001**	156	76.9 (23.6)	0.133	153	**< 0.001**
Cognitive functioning	282	85.6 (19.5)	192	81.3 (22.2)	**0.032**	157	81.6 (23.9)	0.077	153	0.686
Social functioning	272	89.9 (21.3)	191	76.8 (24.5)	**< 0.001**	157	75.4 (28.4)	**< 0.001**	152	0.406
*Symptom scales/items*										
Fatigue	285	28.2 (25.0)	192	39.7 (25.3)	**< 0.001**	157	36.0 (26.4)	**0.003**	153	0.265
Nausea and vomiting	298	3.7 (12.4)	192	9.5 (18.4)	**< 0.001**	157	6.8 (26.4)	**0.038**	153	**0.033**
Pain	280	26.7 (30.5)	192	24.9 (28.7)	0.518	157	22.5 (29.0)	0.155	153	0.448
Dyspnoea	295	22.3 (27.5)	189	22.9 (26.5)	0.802	156	20.1 (26.1)	0.401	149	0.456
Insomnia	295	26.4 (31.2)	191	30.1 (31.6)	0.216	156	34.6 (32.1)	**0.009**	151	0.258
Appetite loss	299	7.4 (18.5)	191	27.2 (32.5)	**< 0.001**	155	16.8 (27.5)	**< 0.001**	151	**< 0.001**
Constipation	298	8.6 (19.6)	190	16.7 (29.6)	**0.001**	155	11.2 (23.2)	0.238	150	**0.014**
Diarrhoea	287	5.0 (14.8)	187	24.4 (31.0)	**< 0.001**	155	20.0 (27.3)	**< 0.001**	147	**0.046**
Financial difficulties	284	8.1 (21.2)	192	8.0 (21.1)	0.954	155	9.7 (25.5)	0.512	151	0.160

^a^Values in the standard population were from sex-specific data for ages 70–79 years given by Michelson et al. (2000)

^b^*P* values for difference between values at baseline and values in the standard population, Significant *P* values are given in bold

^c^*P* values for difference between values at follow-up and values in the standard population

^d^Number of individuals with answers at both baseline and follow-up

^e^*P* values for difference between values at baseline and follow-up

Univariate analysis of differences between the status at baseline and at the 6-month follow-up in the study group as compared with those in the Swedish reference population showed that global health status and emotional function improved in both men and women, and that the scores for constipation and appetite loss improved. At the 6-month follow-up, men had improved social function and improved scores for pain and insomnia, while women had improved scores for nausea/vomiting and diarrhoea (Table 2).

Multiple linear regression analyses (Table 3) of the patients in this study showed that at baseline, patients whose planned surgery included a stoma (with/without bowel resection) had a decrease in 2–4 of 5 functional scales, worse Global Health Status and significantly more symptoms (pain and appetite loss) as compared with patients who underwent surgery without receiving a stoma. The former category of patients also had more fatigue and diarrhoea. Most of these changes persisted at the 6-month follow-up in patients treated with a stoma without bowel resection (Table 3).

**Table 3:** Multiple linear regression analysis of HRQoL of patients with stoma with/without colon resection at baseline and at 6-month follow-up, according to planned stoma before operation, adjusted for age, sex, BMI, smoking, ASA classification, acute/elective surgery and TNM stage

Scale	Baseline					6-month follow-up				
	Stoma with resection (n = 89)^a^		Stoma without resection (n = 14)^a^			Stoma with resection (n = 68)^a^		Stoma without resection (n = 4)^a^		
	*β*	*P*	*β*	*P*	*R*^2^	*β*	*P*	*β*	*P*	*R*^2^
Global health status	− 7.1	**0.025**	− 24.6	**< 0.001**	0.114	1.0	0.782	− 27.0	**0.027**	0.072
*Functional scales*										
Physical function	− 4.2	**0.115**	− 18.9	**0.001**	0.272	− 3.2	0.300	− 11.4	0.269	0.135
Role function	− 9.6	**0.038**	− 33.2	**0.001**	0.123	0.08	0.986	− 22.5	0.209	0.056
Emotional function	− 9.8	**0.001**	− 13.6	0.047	0.094	− 5.9	0.102	− 22.8	0.057	0.114
Cognitive function	− 2.1	0.467	− 2.7	0.679	0.024	− 5.5	0.121	− 24.6	**0.037**	0.073
Social function	− 8.5	**0.015**	− 11.3	0.157	0.058	− 7.9	0.074	− 46.5	**0.002**	0.130
*Symptom scales/items*										
Fatigue	8.4	**0.018**	7.6	0.337	0.079	3.8	0.337	12.4	0.349	0.076
Nausea/vomiting	4.4	0.054	6.4	0.209	0.069	2.2	0.317	30.8	**< 0.001**	0.173
Pain	7.8	**0.044**	22.5	**0.010**	0.073	3.2	0.439	47.7	**< 0.001**	0.103
Dyspnoea	− 0.9	0.807	0.02	0.998	0.151	− 4.1	0.326	− 1.9	0.890	0.091
Insomnia	3.4	0.443	9.3	0.349	0.014	4.3	0.404	16.1	0.342	0.065
Appetite loss	14.0	**0.002**	40.5	**< 0.001**	0.122	− 0.7	0.874	33.5	**0.015**	0.074
Constipation	1.2	0.749	− 5.6	0.513	0.037	− 7.9	**0.017**	18.5	0.083	0.074
Diarrhoea	2.7	0.551	33.4	**0.001**	0.048	2.4	0.570	10.5	0.444	0.080
Financial difficulties	1.7	0.538	2.2	0.721	0.061	2.1	0.588	17.1	0.180	0.134

The highest observed Variance Inflation Factors were 1.404 at baseline and 1.390 at 6-month follow-up, Significant *P* values are given in bold

*ASA* American Society of Anesthesiologists, *BMI* body mass index, *HRQoL* health-related quality of life, *TNM* tumour–node–metastasis

^a^Reference category: No stoma

Table 4 shows the results of multiple linear regression analyses of the change in HRQoL of patients with colon cancer from diagnosis to the 6-month follow-up. Male patients had increased insomnia. Older patients had a greater improvement in emotional and social functions than younger patients. They also showed a greater decrease in nausea/vomiting, appetite loss and diarrhoea at the 6-month follow-up compared with baseline. Smokers showed a greater deterioration in role, emotional and social functions than other patients. They also had a greater increase in fatigue, nausea/vomiting, diarrhoea symptoms and financial difficulties. Patients with colon cancer with higher BMI had decreased physical and cognitive functions, increased nausea/vomiting and pain and more financial difficulties. Patients treated with a stoma without bowel resection had increased levels of nausea/vomiting, pain and financial difficulties at the 6-month follow-up compared with baseline. Type of surgery (right-, left-sided, sigmoid or high anterior resection) did not significantly influence HRQoL (*P* > 0.05).

**Table 4:** Results from multiple linear regression analysis of change in HRQoL from baseline to 6-month follow-up (adjusted for value of each scale at baseline; *β* is the regression coefficient)

Scale	Global health status (QL2)		Physical function (PF2)		Role function (RF2)	
	β (95% CI)	*P*	β (95% CI)	*P*	β (95% CI)	*P*
Male sex	0.54 (− 4.60, 5.68)	0.836	2.84 (− 1.35, 7.04)	0.183	1.33 (− 5.64, 8.30)	0.707
Age (years)	0.08 (− 0.20, 0.36)	0.576	0.08 (− 0.15, 0.31)	0.491	0.19 (− 0.20, 0.57)	0.339
Smoker	− 6.24 (− 15.3, 2.77	0.174	− 6.57 (− 13.8, 0.66)	0.075	− 18.0 (− 30.2, − 5.77)	**0.004**
BMI (kg/m^2^)	− 0.34 (− 0.94, 0.26)	0.261	− 0.53 (− 1.02, − 0.05)	**0.032**	− 0.73 (− 1.55, 0.10)	0.085
ASA 1–2	5.58 (− 0.27, 11.4)	0.062	0.88 (− 4.08, 5.84)	0.727	0.62 (− 7.35, 8.60)	0.878
Emergency	0.40 (− 7.85, 8.65)	0.924	− 0.35 (− 6.96, 6.25)	0.917	8.20 (− 3.14, 19.5)	0.156
TNM I–III	1.61 (− 7.29, 10.5)	0.722	− 0.28 (− 7.53, 6.97)	0.939	0.89 (− 11.5, 13.2)	0.888
Stoma w/ resection	6.37 (− 0.51, 13.3)	0.069	− 0.04 (− 5.46, 5.39)	0.990	2.45 (− 6.74, 11.6)	0.600
Stoma w/o resection	− 7.75 (− 30.49, 14.99)	0.503	3.94 (− 13.96, 21.83)	0.665	− 9.48 (− 43.0, 24.1)	0.578

Scale	Emotional function (EF)		Cognitive function (CF)		Social function (SF)	
	β (95% CI)	*P*	β (95% CI)	*P*	β (95% CI)	*P*
Male sex	2.22 (− 2.74, 7.17)	0.379	1.23 (− 3.64, 6.09)	0.620	− 1.44 (− 7.69, 4.82)	0.651
Age (years)	0.31 (0.03, 0.58)	**0.029**	0.12 (− 0.15, 0.38)	0.378	0.35 (0.001, 0.69)	**0.049**
Smoker	− 9.05 (− 17.6, − 0.45)	**0.039**	− 7.30 (− 15.8, 1.18)	0.091	− 13.9 (− 24.9, − 2.93)	**0.013**
BMI (kg/m^2^)	− 0.22 (− 0.79, 0.35)	0.457	− 0.57 (− 1.13, − 0.003)	**0.049**	− 0.45 (− 1.18, 0.28)	0.228
ASA 1–2	4.37 (− 1.17, 9.91)	0.122	− 1.53 (− 7.01, 3.95)	0.583	2.05 (− 5.02, 9.13)	0.568
Emergency	− 3.77 (− 11.6, 4.02)	0.342	1.02 (− 6.63, 8.66)	0.794	− 2.47 (− 12.5, 7.51)	0.626
TNM I–III	− 3.85 (− 12.3, 4.65)	0.373	1.64 (− 6.77, 10.04)	0.702	− 2.92 (− 13.8, 7.93)	0.597
Stoma w/ resection	− 0.15 (− 6.71, 6.40)	0.963	− 3.63 (− 10.0, 2.75)	0.264	− 3.46 (− 11.8, 4.84)	0.413
Stoma w/o resection	− 7.69 (− 29.07, 13.70)	0.480	− 19.6 (− 40.4, 1.27)	0.066	− 21.5 (− 51.7, 8.79)	0.163

Scale	Fatigue (FA)		Nausea/vomiting (NV)		Pain (PA)	
	β (95% CI)	*P*	β (95% CI)	*P*	β (95% CI)	*P*
Male sex	− 2.01 (− 7.57, 3.56)	0.478	− 0.98 (− 4.26, 2.31)	0.559	− 5.24 (− 11.2, 0.76)	0.087
Age (years)	− 0.30 (− 0.61, 0.00)	0.052	− 0.21 (− 0.39, − 0.03)	**0.024**	0.16 (− 0.17, 0.49)	0.348
Smoker	10.8 (0.99, 20.7)	**0.031**	7.47 (1.77, 13.2)	**0.010**	10.4 (− 0.12, 20.93)	0.053
BMI (kg/m^2^)	0.23 (0.41, 0.88)	0.478	0.52 (0.14, 0.90)	**0.007**	0.80 (0.10, 1.50)	**0.025**
ASA 1–2	− 5.90 (− 12.2, 0.43)	0.068	− 0.16 (− 3.84, 3.52)	0.932	− 1.99 (− 8.77, 4.79)	0.564
Emergency	− 9.61 (− 18.4, − 0.78)	**0.033**	1.52 (− 3.69, 6.74)	0.566	− 8.02 (− 17.5, 1.45)	0.097
TNM I–III	− 8.63 (− 18.3, 1.06)	0.081	− 1.72 (− 7.37, 3.94)	0.551	7.64 (− 2.74, 18.01)	0.148
Stoma w/ resection	− 0.25 (− 7.68, 7.17)	0.947	0.57 (− 3.75, 4.89)	0.795	0.04 (− 7.89, 7.98)	0.992
Stoma w/o resection	− 1.01 (− 25.2, 23.2)	0.935	22.5 (8.09, 39.9)	**0.002**	36.6 (10.6, 62.6)	**0.006**

Scale	Dyspnoea (DY)		Insomnia (SL)		Appetite loss (AP)	
	β (95% CI)	*P*	β (95% CI)	*P*	β (95% CI)	*P*
Male sex	3.81 (− 2.05, 9.66)	0.202	− 12.3 (− 18.8, − 5.82)	**< 0.001**	− 4.14 (− 10.1, 1.82)	0.173
Age (years)	− 0.22 (− 0.54, 0.10)	0.180	− 0.25 (− 0.60, 0.11)	0.172	− 0.41 (− 0.73, − 0.08)	**0.014**
Smoker	1.64 (− 8.79, 12.1)	0.757	9.16 (− 2.22, 20.5)	0.114	5.35 (− 4.99, 15.70)	0.309
BMI (kg/m^2^)	0.63 (− 0.08, 1.33)	0.080	0.64 (− 0.12, 1.39)	0.099	0.23 (− 0.46, 0.91)	0.519
ASA 1–2	− 6.68 (− 13.6, 0.22)	0.058	− 1.63 (− 8.99, 5.74)	0.664	− 3.19 (− 9.88, 3.50)	0.349
Emergency	5.56 (− 3.78, 14.9)	0.242	− 3.58 (− 13.8, 6.69)	0.493	− 2.00 (− 11.4, 7.37)	0.674
TNM I–III	− 5.43 (− 15.5, 4.68)	0.291	4.78 (− 6.49, 16.05)	0.404	6.22 (− 3.99, 16.4)	0.231
Stoma w/ resection	− 5.22 (− 13.03, 2.58)	0.189	0.262 (− 8.30, 8.83)	0.952	− 6.01 (− 13.9, 1.92)	0.137
Stoma w/o resection	− 1.18 (− 26.2, 23.8)	0.926	− 9.11 (− 37.4, 19.1)	0.526	15.4 (− 10.6, 41.3)	0.246

Scale	Constipation (CO)		Diarrhoea (DI)		Financial difficulties (FI)	
	β (95% CI)	*P*	β (95% CI)	*P*	β (95% CI)	*P*
Male sex	− 2.68 (− 7.53, 2.17)	0.277	− 4.40 (− 10.6, 1.81)	0.164	− 0.73 (− 5.30, 3.85)	0.755
Age (years)	− 0.25 (− 0.51, 0.13)	0.062	− 0.49 (− 0.83, − 0.15)	**0.005**	− 0.15 (− 0.41, 0.10)	0.233
Smoker	1.99 (− 6.48, 10.5)	0.643	14.2 (3.32, 25.1)	**0.011**	8.57 (0.43, 16.7)	**0.039**
BMI (kg/m^2^)	− 0.03 (− 0.59, 0.53)	0.922	0.50 (− 0.22, 1.23)	0.173	0.58 (0.05, 1.11)	**0.032**
ASA 1–2	− 5.31 (− 10.8, 0.18)	0.058	− 4.09 (− 11.1, 2.91)	0.251	1.84 (− 3.32, 6.99)	0.483
Emergency	− 1.07 (− 8.88, 6.74)	0.788	0.10 (− 10.1, 10.3)	0.985	1.31 (− 6.05, 8.67)	0.727
TNM I–III	2.98 (− 5.31, 11.3)	0.480	3.33 (− 7.66, 14.3)	0.551	0.21 (− 7.66, 8.08)	0.958
Stoma w/ resection	− 8.38 (− 14.8, − 1.98)	**0.010**	4.11 (− 4.19, 12.2)	0.331	− 0.31 (− 6.36, 5.73)	0.919
Stoma w/o resection	16.5 (− 4.01, 37.0)	0.114	− 18.5 (− 48.1, 11.1)	0.220	20.6 (1.10, 40.0)	**0.038**

The highest observed Variance Inflation Factor was 1.448. R^2^-values: QL2 0.299, PF2 0.215, RF2 0.422, EF 0.280, CF 0.239, SF 0.266, FA 0.354, NV 0.470, PA 0.419, DY 0.346, SL 0.286, AP 0.496, CO 0.596, DI 0.563, FI 0.099, Significant *P* values are given in bold

*ASA* American Society of Anaesthesiologists, *BMI* body mass index, *CI* confidence interval, *HRQoL* health-related quality of life, *TNM* tumour–node–metastasis

## Discussion

The EORTC QLQ-C30 questionnaire was used to investigate HRQoL in patients with colon cancer in this population-based study. The major findings of this study were that patients whose planned surgery included a stoma (with/without bowel resection), those with higher BMI, those with worse health (ASA status 3 and 4) and smokers were at higher risk of a lower HRQoL than the other included patients. Furthermore, this study showed that patients with colon cancer had worse HRQoL than a Swedish reference population both at baseline and at the 6-month follow-up as indicated by changed scores for 3/5 functional (role, emotional and social), and 4/9 symptom (fatigue, nausea/vomiting, appetite loss and diarrhoea) scales.

It is difficult to compare our findings with those of other studies that used a reference population because these studies vary in methodology and the reference values used [17]. However, consistent with our findings, Färkkilä et al. [18], in a study of Finnish patients with colorectal cancer, compared their data with reference data using the EORTC QLQ-C30 questionnaire, and found that pain, fatigue and financial difficulties were the main drivers of poor health. Another study conducted in Germany by Jansen et al. [8] also compared patients with colorectal cancer with control individuals from the general population and showed that diarrhoea and financial difficulties were worse in patients with colorectal cancer.

Several countries have assessed HRQoL in patients with colon cancer, but only one randomized study comparing the effects of open and laparoscopic surgery in Sweden has been published [19]. Apart from that study, there is no published information regarding HRQoL in Swedish patients with colon cancer.

Contradicting results have been presented regarding whether the presence of a stoma in surgically treated patients with colorectal cancer has a negative effect on HRQoL. Most of these studies have been performed on patients with rectal cancer [20]. Notably, the participants in our study completed the questionnaire before they underwent surgery to create a stoma. This implies that it was the patients’ risk factors (as judged by the surgeon) or the advanced stage of their cancers that were related to the observed lower HRQoL in these patients. They might also have had poor expectations of life with a stoma, or it may have been that it was the information that they were to receive a stoma per se that contributed to their poor scores. Although this study included very few patients who were treated with a stoma alone, this group showed significantly worse functional scores and better symptom scores than patients treated with a stoma and bowel resection. Furthermore, a recent study of patients with colon cancer who answered questions postoperatively about what the most important factors for them in life were overall, related to the cancer disease, 78% reported that they considered not having a permanent stoma was the most important factor (76% stated that ‘being cured’ was most important factor) [21].

This study found that HRQoL was not affected by whether the patients underwent right-sided, left-sided or total colectomy (data not shown), and that patients with an advanced tumour stage (TNM IV) did not have significantly worse HRQoL than other patients. This was somewhat surprising. It might be due to a selection bias since the non-included patients had a higher TNM stage. However, 10 of the 14 patients who were treated with a stoma without resection had metastatic disease (TNM IV), and these patients had very low functional scores and high symptom scores, indicating worse HRQoL at both baseline and at the 6-month follow-up.

Our study also found that younger patients had worse emotional and social functional QoL and more bowel problems (nausea/vomiting and diarrhoea) than older patients. This has also been observed by others and suggests that age-matched groups should be used to generate data for HRQoL comparisons [22]. Younger patients might be a more vulnerable group with less coping capacity. They might also view cancer as a greater threat to their lives than older patients.

In our study, worse health status as assessed using ASA status had a negative impact on global health status, physical function, fatigue, dyspnoea and constipation, at both baseline and the 6-month follow-up. These data are also consistent with the results of other studies of colorectal cancer and other cancer types such as head and neck, lung and prostate cancer [23]. In breast cancer, the effect of comorbidity explained most of the variance in nearly all subscales comparing demographic and clinical variables [24].

The data also showed that patients with a higher BMI had worse physical function and more nausea and vomiting, pain and financial difficulties. This observation has also been reported by others [25]. There is increasing evidence that high body weight, often associated with a sedentary lifestyle, is related to impairments in QoL. Considering several different lifestyle factors, Schlesinger et al. [6] found that being non-obese had the strongest association with a high HRQoL, while another study reported decreased HRQoL in Dutch patients with high BMI [25].

The present study also showed that smokers had worse QoL than other patients at the 6-month follow-up compared with baseline. These data are consistent with the findings of studies of the general population [26] and of patients with colorectal cancer [27]. Both these studies reported that current smokers had a higher likelihood of reporting poor physical health, poor mental health and activity limitations. It has also been reported that smoking rate was higher in young survivors of colon cancer and melanoma than in young individuals without cancer. These survivors also had higher age-adjusted smoking rates than survivors of other cancers [28].

### Strengths and limitations

The main strengths of this study were its prospective design and that it was population-based with a fairly high response rate (86%) at 6 months. We also managed to recruit the patients before the start of treatment. The patient data were compared with those from a general Swedish reference population [12]. We also used one of the most widely used validated analysis generic instruments, the EORTC QLQ-C30, and because all patients agreed to be included in the local and national registries, we could also analyse data from non-included patients. In addition, we performed multiple regression analyses of medically important parameters, including lifestyle factors, such as BMI and smoking.

Analysis of data from the non-included patients showed that they had worse health (ASA status 3 and 4), were less frequently treated surgically, had more advanced-stage tumours and had surgical treatment that more often included stoma creation. Thus, presumably, if these patients had been included, our results would have shown an even worse outcome for HRQoL because these non-included patients had evident risk factors for lower QoL [29]. This could also be the case for the patients who were too ill to complete the questionnaire.

Other study limitations were that this was a single-centre study and the reference data for the Swedish general population were collected several years before the present study started [12]. In addition, because no individual data were available for the reference population, comparisons with individuals from the present study had to rely on means, standard deviations and *t*-tests, while medians, interquartile ranges and non-parametric tests could not be evaluated.

Furthermore, we did not analyse social, socio-economic and psychological factors known to influence HRQoL [11]. A small study by Siassi et al. [30] showed that personality affects HRQoL more than clinical variables after a major surgery. We did not include data on the effect of chemotherapy, but other studies have shown that it is not a major factor affecting HRQoL [31–33]. One could argue that had it been an important factor, there would have been an association between TNM stage and HRQoL.

The presence of missing values for some of the analysed variables increased the risk of biased results and limited the generalizability of the results. Likewise, the significant differences in clinical characteristics between included and not-included individuals imply the presence of a selection bias, thus limiting the generalizability of the results. It is probable that the most fragile individuals had a lower response rate at the 6-month follow-up, meaning that the observed changes from baseline to the 6-month follow-up may be somewhat biased. Moreover, notably, the number of patients having a stoma without resection was rather small, n = 14 at baseline and n = 4 at the 6-month follow-up (Table 3), indicating that the observed findings are less robust to possible deviations from the underlying statistical assumptions. Thus, the findings need to be interpreted with some caution. Finally, the sample size limited the possibilities of using other methods for adjustment for confounding than the ones applied, e.g. stratified regression method, since these would have resulted in considerable loss of power and too small subgroups for some categorical variables.

Disease, treatment and patient characteristics are intimately related, and the course of disease and HRQoL will be affected depending on which one of them is dominant. According to our data, patient characteristics such as smoking, high BMI and worse physical health were strongly associated with lower HRQoL, which in turn could be associated with other lifestyle factors and lower socio-economic status. Different forms of social support and lifestyle behaviour recommendations can be of importance and should be explored [10]. Furthermore, treatment with a stoma had a negative impact on HRQoL [34]; therefore, it is important for health care (i.e. surgeons) to facilitate stoma closure when possible.

## Conclusions

In conclusion, this study showed that at baseline, many patients with colon cancer have low HRQoL compared with a refence population, but that HRQoL improved at the 6-month follow-up for patients with non-metastatic disease. We identified both patient- and treatment-related risk groups: younger patients, patients with higher BMI, smokers and patients who underwent stoma surgery. These patients need enhanced treatment, rehabilitation and support. These findings should be used to counsel individual patients in the patients-physician encounter. Moreover, these interventions might also improve their cancer prognosis.

## Data Availability

The datasets used and/or analysed during the current study are available from the corresponding author on reasonable request.

## Abbreviations

HRQoL: Health related quality of life
EORTC QLQ-C30: European Organization for Research and Treatment of Cancer Quality of Life Questionnaire C30
BMI: Body mass index
ASA: American Society of Anesthesiologists
TNM: Tumour-node-metastasis
